# Differential Effects of Cerebellar tDCS on Sequential Mentalizing

**DOI:** 10.1007/s12311-025-01876-1

**Published:** 2025-07-04

**Authors:** Beatriz Catoira, Marco Manzo, Joy de Gabriac, Jens Allaert, Raquel Guiomar, Stefanie De Smet, Natacha Deroost, Marie-Anne Vanderhasselt, Frank Van Overwalle, Chris Baeken

**Affiliations:** 1 Department of Psychiatry (UZ Brussel), Vrije Universiteit Brussel, Brussels, Belgium; 2 Ghent Experimental Psychiatry (GHEP) Lab, Ghent University, Ghent, Belgium; 3 Department of Neuroscience, Reproductive Sciences, and Dentistry, Section of Psychiatry, University School of Medicine Federico II, Naples, Italy; 4 Faculty of Social and Behavioural Sciences, Leiden University, Leiden, the Netherlands; 5 Center for Research in Neuropsychology and Cognitive and Behavioral Intervention, Faculty of Psychology and Educational Sciences, University of Coimbra, Coimbra, Portugal; 6 Department of Psychology and Center for Neuroscience, Vrije Universiteit Brussel, Brussels, Belgium

**Keywords:** Cerebellum, tDCS, Mentalizing, Picture sequencing, Social cognition

## Abstract

The cerebellum has been increasingly recognized for its role in social cognition, particularly in mentalizing processes. A way to measure mentalizing is the picture sequencing task, a well-established measure of social action sequencing during mentalizing of other’s beliefs. Recent studies have shown that cerebellar transcranial direct current stimulation can affect social sequence processing in adults, however, the effects of different types of stimulation remain unclear. Therefore, in this study, we examined the effects of a novel and more focal montage of cerebellar tDCS on the picture sequencing task in healthy adults. Using a within-participant design, 35 participants completed three sessions in which they underwent anodal, cathodal, and sham stimulation (in a counterbalanced order). Results revealed that participants were consistently slower on sequences that required complex mentalizing compared to well-known social situations and non-social events. Anodal tDCS significantly speeded up reaction times from the second session (indicating an improvement in performance), sham tDCS showed the same improvement in the third session (indicating general improved familiarity with the task), while cathodal tDCS did not change performance. This interaction between stimulation type and session suggests that anodal tDCS may accelerate sequence learning, while cathodal tDCS may inhibit it. Accuracy results reflected a similar pattern, with improvements over time driven by the stimulation-learning interaction. In conclusion, cerebellar tDCS modulates performance with anodal stimulation enhancing processing speed and learning. More importantly, the interaction between the different types of stimulation and learning reinforces the importance of the cerebellum in social learning processes.

## Introduction

The cerebellum has emerged as a critical structure in understanding not only motor coordination [29], but also the cognitive and affective processes that shape how we think, feel, and interact with others. Although past research had already revealed its involvement in cognitive processes and emotional regulation [38], more recent findings have consolidated the key role that the cerebellum plays in social processes [23, 39, 46]. Some of these social processes include mental state attribution and interpersonal interactions [4], action prediction based on personality traits [16], implicit social learning [27], and mentalizing [41].

Mentalizing is the ability to understand and interpret the mental states (such as desires, intentions and beliefs) of others [9]. Functional MRI studies have further provided evidence for the cerebellum’s involvement in mentalizing tasks, particularly those requiring complex social reasoning, such as understanding personality traits or hypothetical situations [42]. Specifically, the posterior part of the cerebellum has been linked to mentalizing processes [20, 26].

Beyond cerebellar activation during social tasks, the cerebellum has also shown functional connectivity with multiple regions of the mentalizing network [47], which encompasses critical cortical regions involved in processing social information, such as the medial prefrontal cortex and the temporoparietal junction. This connection suggests that the cerebellum plays a supportive role in facilitating the efficiency of cognitive processes related to social understanding and interpersonal dynamics [1, 22, 30]. With its extensive connectivity to cortical regions involved in social processing, the cerebellum may be a key player in constructing cognitive frameworks for mentalizing [14].

A widely used task to assess mentalizing is the Picture Sequencing task [24]. This task has been proven to activate the posterior cerebellum [20] and other areas of the mentalizing network [3, 47]. This task was first developed by Langdon & Coltheart [24] and it requires participants to generate the correct chronological order of an event depicted in four pictures. The original version of the task focuses on false belief events. False beliefs sequences depict a story in which something happens (for example an object is misplaced) while the main character of the story is not present, and this leads the main character of the story to have a false belief (such as trying to find the object in the previous location). The participant must be able to separate their own knowledge (knowing the correct location of the object) from the knowledge of the main character. This original version also included as contrasts non-social events and well-known social events (such as paying for groceries in a supermarket). A later version of this task was expanded to include novel events that depict true belief sequences (without any false beliefs involved; Heleven et al. [20]). The complexity of false and true beliefs is demonstrated by an increase in reaction times when compared to social scripts and mechanical events in healthy participants [3, 20, 43].

The picture sequencing task has also been used to assess mentalizing skills in clinical populations. For example, autistic individuals displayed significantly longer reaction times (compared to neurotypical controls) in those sequences that required an understanding of social situations (such as false beliefs), however, there were no differences in non-social events [18]. A similar pattern was also observed in cerebellar [43] and bipolar disorder patients [48] patients. Furthermore, healthy individuals with high traits in schizotypy seem also to perform worse in false beliefs when performing this task [24].

Recently, several studies have shown that cerebellar neurostimulation can affect social sequence processing in adults [3, 19, 44]. Transcranial direct current stimulation (tDCS) is a non-invasive brain stimulation technique that modulates neuronal excitability by delivering a weak direct current through electrodes placed on the scalp [49]. According to early studies in motor functions of the cerebellum [10], anodal tDCS is believed to increase cerebellar activity, while cathodal tDCS can decrease cerebellar function. However, there seems to be no consensus regarding social tasks, with some studies finding improvements after both anodal and cathodal tDCS [6], others demonstrating that tDCS over the cerebellum decreases activation in areas of the brain associated with social cognition, leading to decreased task performance in sequencing social events [3] and other studies finding positive effects of anodal tDCS [5, 15]. Furthermore, a study that used two similar montages to target the anterior sensorimotor cerebellum and the right posterolateral cerebellum proved that the effects of cerebellar tDCS seem to be highly dependent on the positioning of the tDCS electrodes [36]. As such, the variety of montages and inconsistent findings in the literature regarding polarity-dependent effects make it challenging to draw definitive conclusions [31]. By targeting specific cerebellar circuits, tDCS could potentially improve social cognition and interpersonal communication, thereby promoting better social integration and functioning [6]. Therefore, further exploration of cerebellar tDCS could pave the way for innovative strategies aimed at improving social skills in both clinical and non-clinical populations [7, 37]. The application of cerebellar tDCS for the enhancement of social skills may nevertheless hold a promising potential for therapeutic interventions in populations with social difficulties [45].

Consequently, in this study, we aimed to elucidate the effects of a novel montage for cerebellar tDCS on the picture sequencing task. In order to more accurately target the right posterior cerebellum, we applied a new montage more focal than commonly used montages. We used a within subject design, in which we included anodal, cathodal, and sham stimulation. Our hypothesis was that cerebellar stimulation can have facilitatory (anodal) or inhibitory (cathodal) effects on task performance when compared to sham.

## Materials and Methods

### Participants

Recruitment was done via the distribution of flyers on social media and university boards. In order to ensure safety and the recruitment of healthy participants with no symptoms of mental disorders as well as clinical scores in autism traits (> 32), online screening included a tDCS safety checklist, the modified miniscreen (Mini International Neuropsychiatric Interview, version 5.0.0; Lecrubier et al. [25]; Dutch version by Overbeek et al., [32] and the Autism Quotient (AQ, [2, 21] questionnaires, we included a total of 36 healthy participants (ages between 18 and 41 *x̄* = 26 years, *SD* = 5.81, 22 females and 14 males). One participant was excluded from the analysis for not completing all three experimental sessions.

### Study Design

**Fig. 1:**
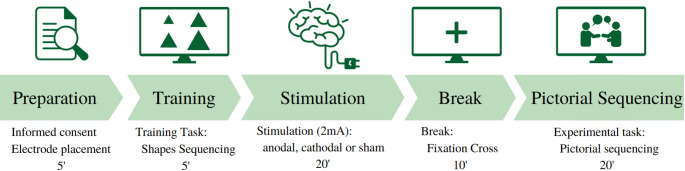
Experimental protocol

The three experimental sessions (anodal, cathodal, and sham) lasted for one hour and they were scheduled one week apart. At the beginning of each session participants gave their informed consent and were explained the outline of the experiment (Fig. 1). Then we continued with the placement of the tDCS electrodes. After that, participants completed a short (5 min) training task, followed by 20 min of stimulation, 10 min of rest and ending with the Picture Sequencing task (approximately 15 min). At the end of the third session, they were asked to guess the type of stimulation that they received on each session to ensure blinding, received monetary compensation and were debriefed about the experiment. This study was approved by the Medical Ethics Committee of the UZGent University Hospital in accordance with the Declaration of Helsinki. Clinical trial number: not applicable.

### tDCS Protocol

**Fig. 2:**
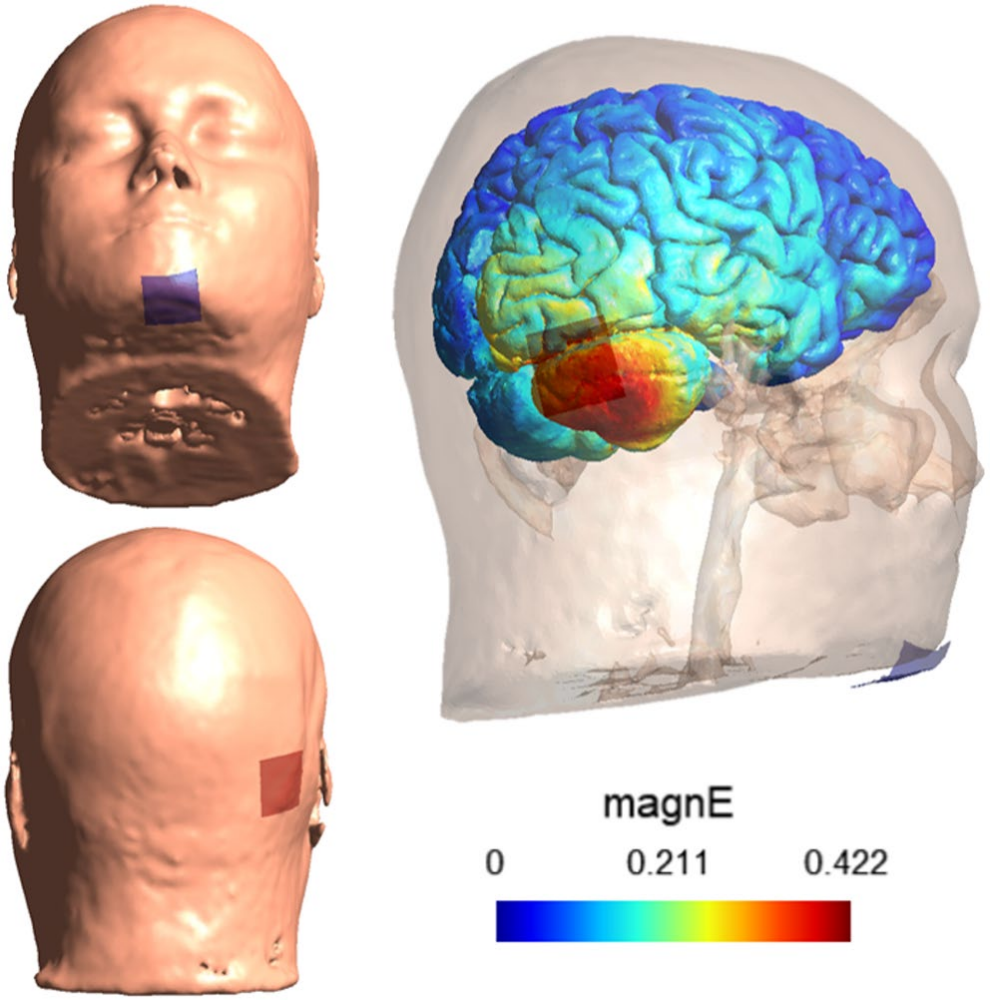
tDCS montage. The anode (indicated by a red patch) was located over the right posterior cerebellum) and the cathode (blue patch) under the chin. On the right, we can observe the electrical distribution of the electric field magnitude (magnE, in Volts per meter- V/m) on the example head Ernie, provided by SimNibs

Three different types of stimulation were used: anodal, cathodal and sham. The order of the three types of the stimulation was counterbalanced. Participants were blinded and they were only informed of the order of the stimulation at the end of the last session. Stimulation was applied via two 3 × 3 cm carbon rubber electrodes with a layer of 1–2 mm of conductive gel (Ten20 Paste, Weaver). The electrodes were fixed using two rubber bands. Following previous research in our lab [13] we used the PO10-chin montage in order to maximize the locality of stimulation over the right posterior cerebellum (Fig. 2). The chin electrode was located at the base of the chin, and PO10 was positioned by measuring a 10% of the distance between Iz and Nz, crossing through A10.

The stimulation was applied using a DC-Stimulator Plus (Model 0021 neuroCare, Ilmenau, Germany). Stimulation lasted 20 min with a ramp up and a ramp down of 30 s each. The intensity was kept at 2 mA for anodal and cathodal stimulation, for the sham stimulation a ramp up and a ramp down of 30 s were applied at the beginning and at the end of the 20 min (with no stimulation in-between) in order to mimic the same sensations produced by the other conditions [8].

### Task

The original picture sequencing task was developed by Langdon and Coltheart [24] and extended with a true belief condition by Heleven et al. [20]. This task includes four different conditions. The non-social conditions depict mechanical events that show purely action-reaction sequences without the involvement of human agents. Social scripts include well-known routine human actions such as brushing teeth or buying at a supermarket. True beliefs are social sequences that present novel situations with social components. False beliefs are also novel situations but that include an action that occurs unbeknown to the main character in the sequence and requires that the participant understands that the main character has less information about that action than an external observer.

Each trial presented four pictures in a random order. Participants reconstructed the sequence by selecting positions 1–3 in order via four button presses: the first three presses assigned pictures to the first, second, and third sequence positions, respectively. Since only one picture remained for the fourth position, the final press either confirmed (correct sequence) or canceled (incorrect sequence) the trial. Participants used four labeled buttons (1–4) to make selections, with visual feedback indicating chosen positions.

Since previous experiments using this task demonstrated that there is a learning effect in which participants become gradually faster [3], a training task was developed with the exact same mechanics in order to train the participants in the button pressed and sequencing dynamics of the task before performing the Picture Sequencing task. The training task consisted of four pictures with geometric shapes of different sizes. The goal was to order the shapes from smallest to biggest.

Both tasks were built using Psychopy functions (Psychopy version 2022.1.1 [33], on a Python script (Python 3.7.11, Spyder). The training task included 2 practice trials followed by 8 experimental trials. The Picture Sequencing task began each session with two practice trials, followed by 28 experimental trials (comprising 7 trials per condition across the four conditions: false belief, true belief, social script, and mechanical). Stimuli were randomized such that both the selection and order of trials varied across sessions. With three sessions in total, this resulted in 84 experimental trials per participant; a unique picture sequence was used for each trial.

### Behavioral Analysis

The picture sequencing task was analyzed on 3 outcome measurements: accuracy (following the original scoring from Langdon and Coltheart [24], reaction time until the 1st button press (RT1) and reaction time until confirmation or cancellation of the sequence (RTTotal). All three measures were analyzed using R 4.2.3 and RStudio/2022.12.0 + 353 (lmerTest package).

A Mixed Model Analysis was performed including Subject as random intercept in order to take into account inter participant variability. The Fixed effects included Picture Condition (false belief, true belief, social script and mechanical), Stimulation (anodal, cathodal and sham) and Session (one, two or three). For all three outcomes the formula was:

Dependent Variable ~ PictureCondition*Stimulation*Session + (1|participant).

We tested several generalized linear mixed models (GLMMs) to accommodate different data distributions: two Gamma models (identity/log link), an Inverse Gaussian model (log link), and a Gaussian model (log link). These distributions were selected to accommodate different data characteristics: Gamma and Inverse Gaussian for positive continuous outcomes (common in reaction time analyses), and Gaussian for symmetric error structures. The model with the best fit was selected according to the Akaike Information Criterion. Follow-up pairwise comparisons had their p-values corrected for multiple comparisons using False Discovery Rate.

## Results

A mixed-effects analysis of variance (ANOVA) was conducted to evaluate the effect of Picture Condition, Stimulation, and Session on three outcomes: RT1, RTTotal and Accuracy (ACC).

### Reaction time to First Button Press (RT1)

The results revealed a significant main effect of Picture Condition 𝜒²(3, *N* = 35) = 1049.306, *p* < 0.001; Stimulation 𝜒²(2, *N* = 35) = 6.217, *p* < 0.05 and Session 𝜒²(2, *N* = 35) = 13.858, *p* < 0.001. Additionally, a significant interaction was observed between Stimulation and Session 𝜒²(4, *N* = 35) = 20.315, *p* < 0.001, indicating that the effect of stimulation varied across sessions.

**Fig. 3:**
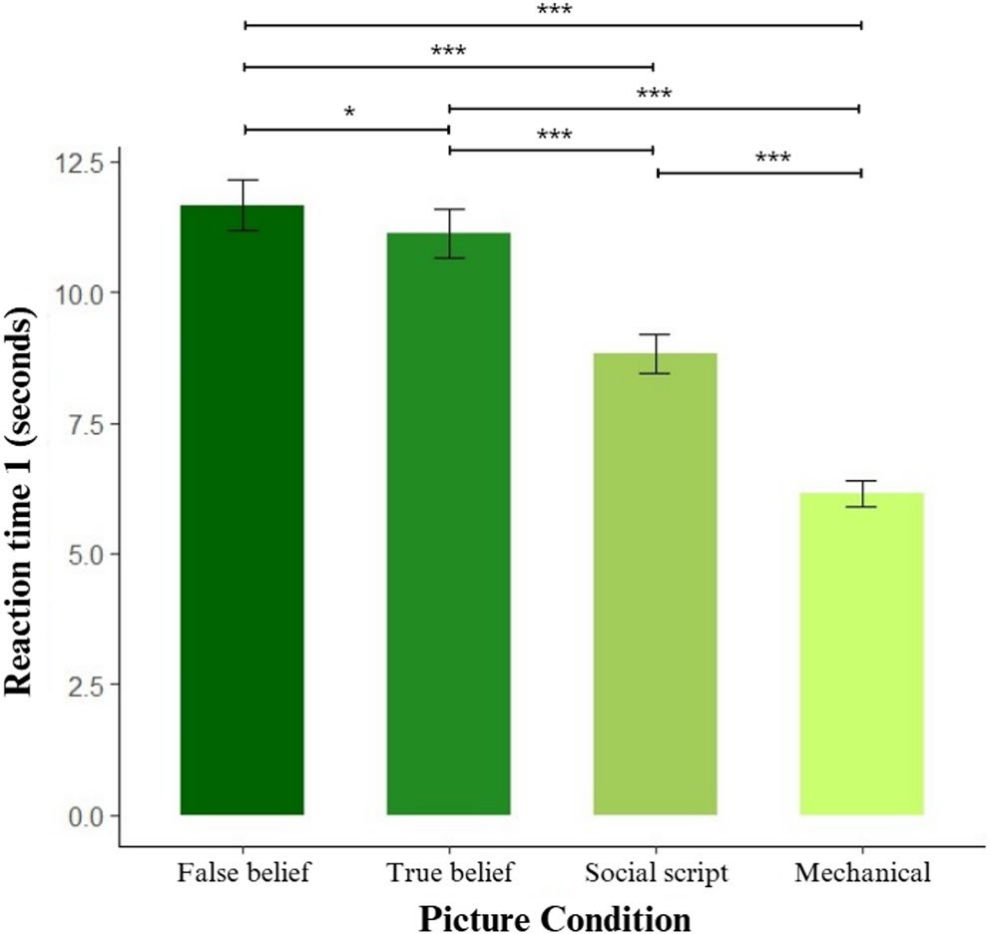
Main effect of Picture Condition on RT1. Notes: Significance levels: * *p* < 0.05, *** *p* < 0.001. Error bars display the standard error. Participants were significantly slower at false belief sequences compared to true belief (𝛽 = 0.048, *SE* = 0.022, *z* = 2.182, *p* < 0.05), social script (𝛽 = 0.279, *SE* = 0.022, *z* = 12.726, *p* < 0.001) and mechanical sequences (𝛽 = 0.641, *SE* = 0.0022, *z* = 28.993, *p* < 0.001). True belief sequences were also slower than social script (𝛽 = 0.231, *SE* = 0.022, *z* = 10.568, *p* < 0.001) and mechanical sequences (𝛽 = 0.593, *SE* = 0.022, *z* = 26.879, *p* < 0.001), and social scripts were slower than mechanical sequences (𝛽 = 0.362, *SE* = 0.022, *z* = 16.422, *p* < 0.001)

As shown in Fig. 3, post-hoc pairwise comparisons revealed that participants performed at significantly faster speeds in RT1 when moving from complex social to non-social picture conditions (false belief, true belief, social script and mechanical). The main effect of Stimulation was driven by a significant difference between anodal and sham stimulation, with participants being overall faster after anodal stimulation (𝛽 = -0.047, *SE* = 0.019, *z* = -2.435, *p* < 0.05). The main effect of Session reflected a learning effect, with faster responses in session 3 compared to session 1 (𝛽 = 0.071, *SE* = 0.019, *z* = 3.709, *p* < 0.001).

**Fig. 4:**
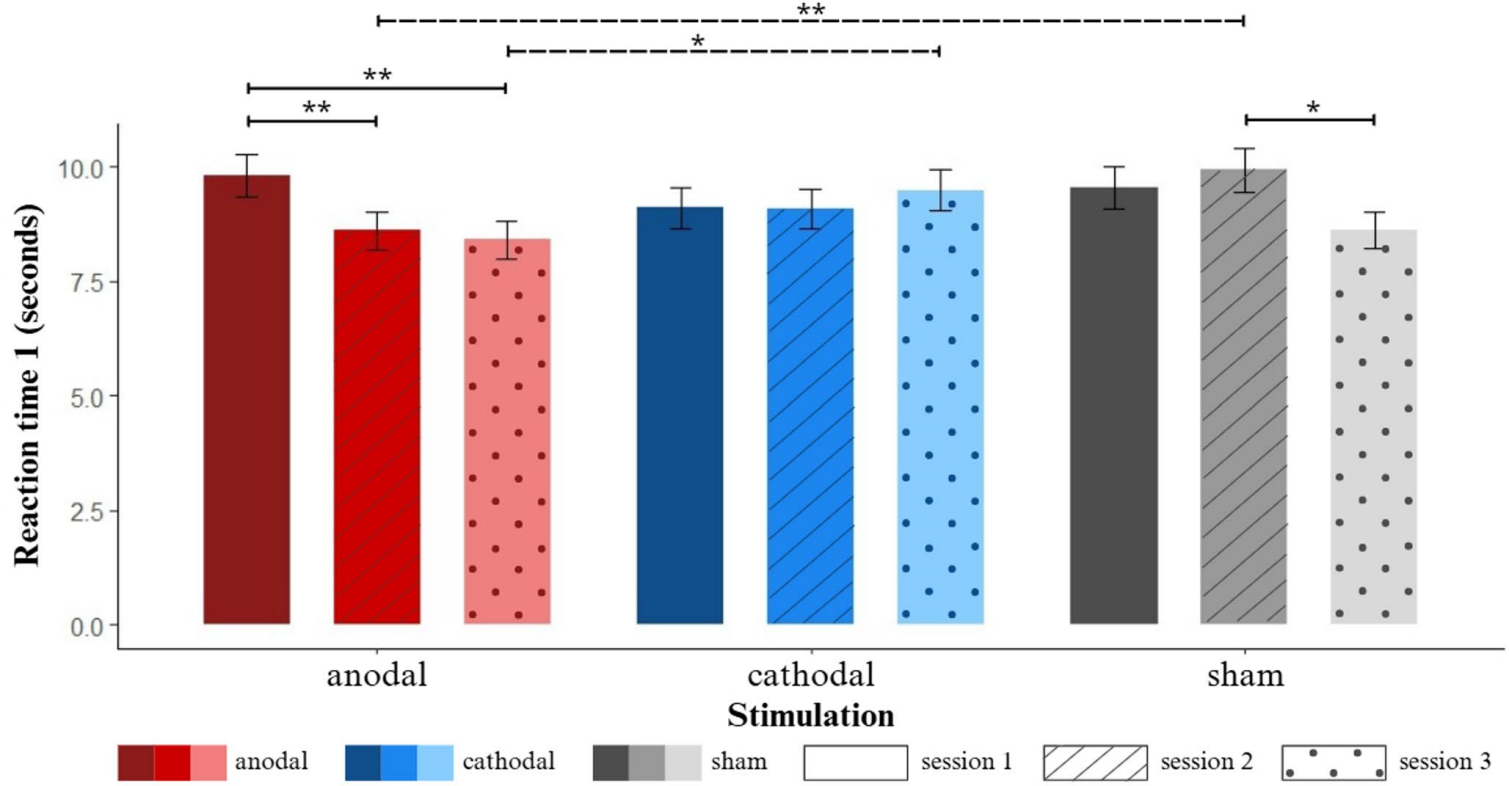
Interaction Stimulation x Session on RT1. Notes: Significance levels: * *p* < 0.05, ** *p* < 0.01. Error bars display the standard error. Solid lines indicate significant differences between sessions within the same type of stimulation, dashed lines indicate significant differences within the same session, between types of stimulation

However, these main effects must be interpreted in light of the Stimulation × Session interaction, displayed in Fig. 4. FDR-corrected pairwise comparisons showed that in session 2, anodal stimulation led to significantly faster performance compared to sham (𝛽 = -0.143, *SE* = 0.043, *z* = -3.346, *p* < 0.01). In session 3, likewise, anodal stimulation led to significantly faster performance compared to cathodal stimulation (𝛽 = -0.121, *SE =* 0.044, *z* = -2.764, *p* < 0.05). Between-session comparisons showed that for the sham condition performance in session 3 was significantly faster than session 2 (𝛽 = 0.14, *SE =* 0.042, *z* = 3.378, *p* < 0.01). Furthermore this improvement across sessions was also observed after anodal stimulation in which both session 3 (𝛽 = 1.155, *SE =* 0.043, *z* = 3.59, *p* < 0.01) and 2 (𝛽 = 0.132, *SE =* 0.042, *z* = 3.118, *p* < 0.01) were faster than session 1. No significant session differences were observed for cathodal stimulation.

### Reaction Time to Sequence Confirmation or Cancellation (RTTotal)

For RTTotal, the mixed-effects ANOVA showed a significant main effect of Picture Condition 𝜒²(3, *N* = 35) = 855.516, *p* < 0.001, and Session 𝜒²(2, *N* = 35) = 36.449, *p* < 0.001. A significant interaction was also found between Stimulation and Session 𝜒²(4, *N* = 35) = 10.828, *p* < 0.05.

In line with the results obtained with RT1, we also observed significant differences between sequences involving all picture conditions (see Table 1), except that there was no significant difference between false and true belief sequences. As seen before, a significant learning effect was observed (see Table 1) in which subsequent sessions were faster than the previous session.

**Table 1:** Main effects of picture condition and session in RTTot

Main effects		𝛽	SE	z	*p*
*Picture Condition*					
	False belief - Social script	0.229	0.017	13.205	< 0.001
	False belief - Mechanical	0.44	0.017	25.521	< 0.001
	True belief - Social script	0.204	0.017	11.825	< 0.001
	True belief - Mechanical	0.415	0.017	24.196	< 0.001
	Social Script - Mechanical	0.212	0.017	12.359	< 0.001
*Session*					
	Session 1 - Session 2	0.048	0.015	3.18	< 0.01
	Session 1 - Session 3	0.091	0.015	6.037	< 0.001
	Session 2 - Session 3	0.043	0.015	2.901	< 0.01

Regarding the interaction between Stimulation and Session, in session 3, anodal stimulation lead to significantly faster responses than cathodal stimulation (𝛽 = -0.096, *SE* = 0.034, *z* = 4.292, *p* < 0.05), and also significantly faster responses than anodal in session 1 (𝛽 = 0.146, *SE* = 0.034, *z* = -2.794, *p* < 0.001). Session 3 also led to faster responses than session 2 for sham (𝛽 = 0.137, *SE* = 0.035, *z* = 3.961, *p* < 0.001), consistent with the learning effect shown in RT1 for anodal and sham conditions.

### Accuracy (ACC)

Analysis of accuracy demonstrated a significant main effect of Picture Condition 𝜒²(3, *N* = 35) = 45.369, *p* < 0.001 and Session 𝜒²(2, *N* = 35) = 10.472, *p* < 0.01. Furthermore, significant interactions were observed between Picture Condition and Session 𝜒²(6, *N* = 35) = 14.572, *p* < 0.05; and between Stimulation and Session 𝜒²(4, *N* = 35) = 17.263, *p* < 0.01.

For the main effect of picture condition, the same pattern as RT1 and RTTotal was observed, with participants having the best performance in the non-social cognition and worsening with the increase of complexity of the social conditions. Participants were significantly less accurate in false beliefs compared to true beliefs (𝛽 = -0.047, *SE* = 0.013, *z* = -3.7, *p* < 0.001), social script (𝛽 = -0.051, *SE* = 0.013, *z* = -4.073, *p* < 0.001) and mechanical sequences (𝛽 = -0.083, *SE* = 0.012, *z* = -6.716, *p* < 0.001). Likewise, they performed significantly less accurately to true beliefs compared to mechanical sequences (𝛽 = 0.036, *SE* = 0.012, *z* = 3.005, *p* < 0.01) and to social scripts compared to mechanical sequences (𝛽 = 0.032, *SE* = 0.012, *z* = 2.668, *p* < 0.01). In line with RT1 and RTTotal, participants performed more accurately in session 3 compared to session 1 (𝛽 = -0.035, *SE* = 0.011, *z* = -3.222, *p* < 0.01).

Pairwise comparisons of the interaction between Stimulation and Session revealed that during session 1 participants who received anodal stimulation performed more accurately than those who received sham (𝛽 = 0.071, *SE* = 0.022, *z* = 3.239, *p* < 0.05). Additionally, participants who received sham in the first session were significantly less accurate than those who received sham in the third session (𝛽 = -0.096, *SE* = 0.022, *z* = -4.347, *p* < 0.001).

**Fig. 5:**
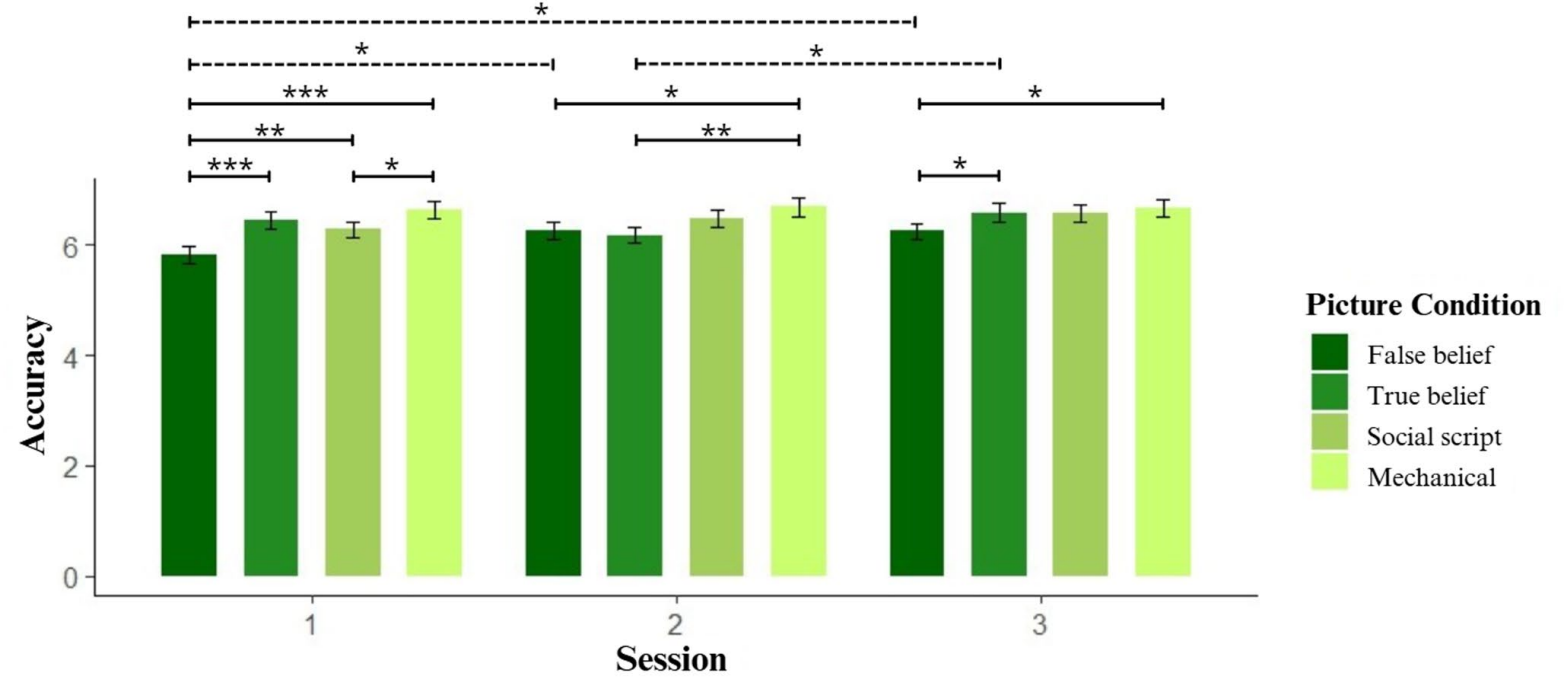
Interaction picture condition x session on accuracy. Notes: Significance levels: * *p* < 0.05, ** *p* < 0.01, *** *p* < 0.001. Error bars display the standard error. Solid lines indicate significant differences between picture conditions within a session, dashed lines indicate significant differences within picture conditions, between sessions. In session 1, participants responded with significantly lower accuracy in false beliefs compared to true beliefs (𝛽 = -0.099, *SE* = 0.023, *z* = -4.353, *p* < 0.001), social scripts (𝛽 = -0.074, *SE* = 0.023, *z* = -3.21, *p* < 0.01) and mechanical (𝛽 = -0.124, *SE* = 0.023, *z* = -5.52, *p* < 0.001) sequences. Also in session 1, participants were less accurate in social script compared to mechanical sequences (𝛽 = -0.05, *SE* = 0.021, *z* = -2.376, *p* < 0.05). In session 2, participants were less accurate in false belief sequences compared to mechanical (𝛽 = -0.064, *SE* = 0.021, *z* = -3.042, *p* < 0.05) and in true belief compared to mechanical (𝛽 = -0.073, *SE* = 0.021, *z* = -3.448, *p* < 0.01). In session 3, participants were less accurate in false beliefs compared to true beliefs (𝛽 = -0.051, *SE* = 0.021, *z* = -2.38, *p* < 0.05) and mechanical (𝛽 = -0.062, *SE* = 0.021, *z* = -2.97, *p* < 0.05) conditions. Furthermore, accuracy in false belief sequences was lower in session 1 compared to session 2 (𝛽 = -0.068, *SE* = 0.023, *z* = -2.94, *p* < 0.05) and session 3 (𝛽 = -0.069, *SE* = 0.023, *z* = -2.99, *p* < 0.05), indicated by dashed lines in Fig. 5. Accuracy in true belief sequences was significantly higher in the session 3 compared to session 2 (𝛽 = -0.061, *SE* = 0.022, *z* = -2.828, *p* < 0.05)

Regarding the interaction between Picture Condition and Session, as depicted in Fig. 5, we observed that accuracy in mechanical and social script sequences was always high, while accuracy for false and true beliefs improved over time, showing a similar pattern as in reaction times. Specifically, in session 1 and 2 participants perform better in mechanical and social script and worse in false and true beliefs. This pattern changes in session 3 where all conditions, except false belief, achieve peak accuracy.

## Discussion

This study aimed to investigate the effects of a novel cerebellar tDCS montage (PO10-chin) on mentalizing. We included anodal, cathodal, and sham stimulation to determine whether they would have a facilitatory or inhibitory effect on social sequencing. Across three sessions, participants received each of the three types of stimulation before performing the picture sequencing task. This task included different levels of social mentalizing (false beliefs, true beliefs, and social scripts) and non-mentalizing sequences (mechanical events). Anodal and Sham stimulation exhibited a learning effect, leading participants to respond with faster reaction times and higher accuracy over time. This learning effect was present from session 2 for anodal tDCS and it became present in session 3 for sham. No learning was observed with cathodal tDCS.

Consistent with previous research, we observed an improvement in task performance across sessions [3, 19] and anodal stimulation improved overall performance [12, 15]. Furthermore, by session 3, participants who received anodal stimulation outperformed participants who received cathodal stimulation. The differential timing of learning effects across stimulation conditions (where anodal tDCS led to earlier improvements in session 2, sham-related gains only emerged in session 3, and cathodal tDCS did not change performance) suggests polarity-specific modulation of cerebellar contributions to learning. One possibility is that prior exposure to the task may prime cerebellar circuits, allowing anodal stimulation to facilitate sequence-related performance once basic task demands are familiar, consistent with procedural learning. In contrast, the gradual improvement observed under sham is likely attributable to general task-related practice effects, or familiarity with the task. Although participants who received cathodal stimulation in the first session were initially faster than those in the sham condition, those who received cathodal stimulation in session 2 and 3 did not show this typical learning effect. This aligns with other research suggesting that cathodal tDCS may hinder task learning through its hypothesized inhibitory nature [12, 28, 35].

Unlike Ferrucci et al. [6], who found no polarity-specific effects on an emotion recognition task, we observed different effects between anodal and cathodal stimulation. These differing results may be due to task variation and the use of our more focal montage (smaller electrodes). Our findings also contrast with the absence of behavioural effects reported by D’Mello et al. [5], although their study observed increased cerebellar activation after tDCS, they hypothesized that ceiling effects in reaction times prevented measurable behavioural changes. The reduced likelihood of ceiling effects in more complex tasks, such as our picture sequencing task, highlights the critical role of task demands in identifying cerebellar tDCS effects. While a recent meta-analysis suggests that anodal tDCS generally improves performance in social tasks when no learning effects are present [40], other studies highlight that the effects of anodal tDCS may depend on anodal placement, with more central or lateralized positioning leading to either facilitatory or mixed effects [34].

Across stimulation conditions, our participants responded more slowly to sequences involving high-level mentalizing (i.e., false and true beliefs). Furthermore, we observed a significant difference between false and true beliefs when analysing RT1. This finding had not yet been reported in previous non-invasive stimulation studies [3, 20], but seems consistent with the typical finding in neuroimaging that false beliefs often elicit stronger activation than true beliefs [41],. It provides further evidence that processing false belief stories involves more complex cognitive processes, requiring more time for people to process and sequence the information as compared to true beliefs. However, this difference disappeared in RTTotal, indicating that this difference in reaction time fades as participants complete the full sequence. This suggests that RT1 may be a more sensitive measure of mentalizing processing demands, while RTTotal may capture overall task performance, which improves with practice perhaps due to motor learning [17]. Consequently, this improvement in the motor aspects of the task might be the cause behind the stronger learning effect in RTTotal compared to RT1, and this could be irrespective of stimulation condition. Our accuracy analysis further supported the distinction between false and true beliefs: initially, false belief sequences were completed with lower accuracy than other conditions, but performance improved over time. Social scripts and mechanical events showed no learning effect, likely due to a ceiling effect.

Although our study has certain strengths, such as the improved cerebellar tDCS montage, several limitations should be discussed. First, our design was constrained by the pictorial sequencing task, which has a limited number of trials. While this task has been expanded from the original four trials per condition [24] to seven (for each session) in the current study, this may still be insufficient for a complex within-subject design involving multiple sessions and stimulation types. Increasing the number of trials could have enhanced statistical power, but this may also lead to participant fatigue, particularly for sequences with longer reaction times. Second, our within-subject design, while useful for examining the interaction between stimulation and session, also presented challenges. A between-subject design could improve statistical power but would not adequately capture session-specific learning effects observed in this and previous research. Another key limitation is that we applied stimulation before the task rather than during task performance, which may not fully capture the dynamic effects of tDCS on cognitive processing.

Future studies should integrate neuroimaging techniques, such as fMRI or EEG, to directly measure changes in cerebellar neuronal changes induced by tDCS. Combining behavioral and neurophysiological data would provide more precise insights into how cerebellar stimulation influences social cognition and sequencing tasks. Additionally, task design should be refined to better balance the number of trials and participant fatigue. While increasing the number of trials may improve statistical power, alternative approaches—such as using shorter or adaptive sequencing tasks—could help optimize measurement sensitivity without excessive cognitive load. Future research should also explore the impact of stimulation timing. Comparing the effects of pre-task vs. online stimulation (i.e., applying tDCS during task execution) may reveal whether concurrent stimulation enhances cognitive processing differently than stimulation applied beforehand.

## Conclusion

Our results contribute to the growing body of evidence demonstrating the cerebellum’s role in social cognition. Modulating the activity of the right posterior cerebellum through tDCS influences both task performance and learning, reinforcing the cerebellum’s key role in social learning processes. While our findings suggest that anodal tDCS enhance task performance, the observed variability in effects highlights the need for further investigation into the interplay between stimulation parameters, task demands, and individual differences. Future research should continue refining stimulation protocols to optimize cerebellar tDCS as a tool for modulating social cognitive functions.

## Data Availability

No datasets were generated or analysed during the current study.
